# Biochemical Properties of Human D-amino Acid Oxidase Variants and Their Potential Significance in Pathologies

**DOI:** 10.3389/fmolb.2018.00055

**Published:** 2018-06-12

**Authors:** Silvia Sacchi, Pamela Cappelletti, Giulia Murtas

**Affiliations:** ^1^Dipartimento di Biotecnologie e Scienze della Vita, Università degli Studi dell'Insubria, Varese, Italy; ^2^The Protein Factory, Politecnico di Milano and Università degli Studi dell'Insubria, Milan, Italy

**Keywords:** D-amino acid oxidase, D-amino acids, D-serine levels, protein variants, structural-functional properties, protein conformation, human pathologies

## Abstract

The stereoselective flavoenzyme D-amino acid oxidase (DAAO) catalyzes the oxidative deamination of neutral and polar D-amino acids producing the corresponding α-keto acids, ammonia, and hydrogen peroxide. Despite its peculiar and atypical substrates, DAAO is widespread expressed in most eukaryotic organisms. In mammals (and humans in particular), DAAO is involved in relevant physiological processes ranging from D-amino acid detoxification in kidney to neurotransmission in the central nervous system, where DAAO is responsible of the catabolism of D-serine, a key endogenous co-agonist of N-methyl-D-aspartate receptors. Recently, structural and functional studies have brought to the fore the distinctive biochemical properties of human DAAO (hDAAO). It appears to have evolved to allow a strict regulation of its activity, so that the enzyme can finely control the concentration of substrates (such as D-serine in the brain) without yielding to an excessive production of hydrogen peroxide, a potentially toxic reactive oxygen species (ROS). Indeed, dysregulation in D-serine metabolism, likely resulting from altered levels of hDAAO expression and activity, has been implicated in several pathologies, ranging from renal disease to neurological, neurodegenerative, and psychiatric disorders. Only one mutation in *DAO* gene was unequivocally associated to a human disease. However, several single nucleotide polymorphisms (SNPs) are reported in the database and the biochemical characterization of the corresponding recombinant hDAAO variants is of great interest for investigating the effect of mutations. Here we reviewed recently published data focusing on the modifications of the structural and functional properties induced by amino acid substitutions encoded by confirmed SNPs and on their effect on D-serine cellular levels. The potential significance of the different hDAAO variants in human pathologies will be also discussed.

## Introduction

**Figure 1:**
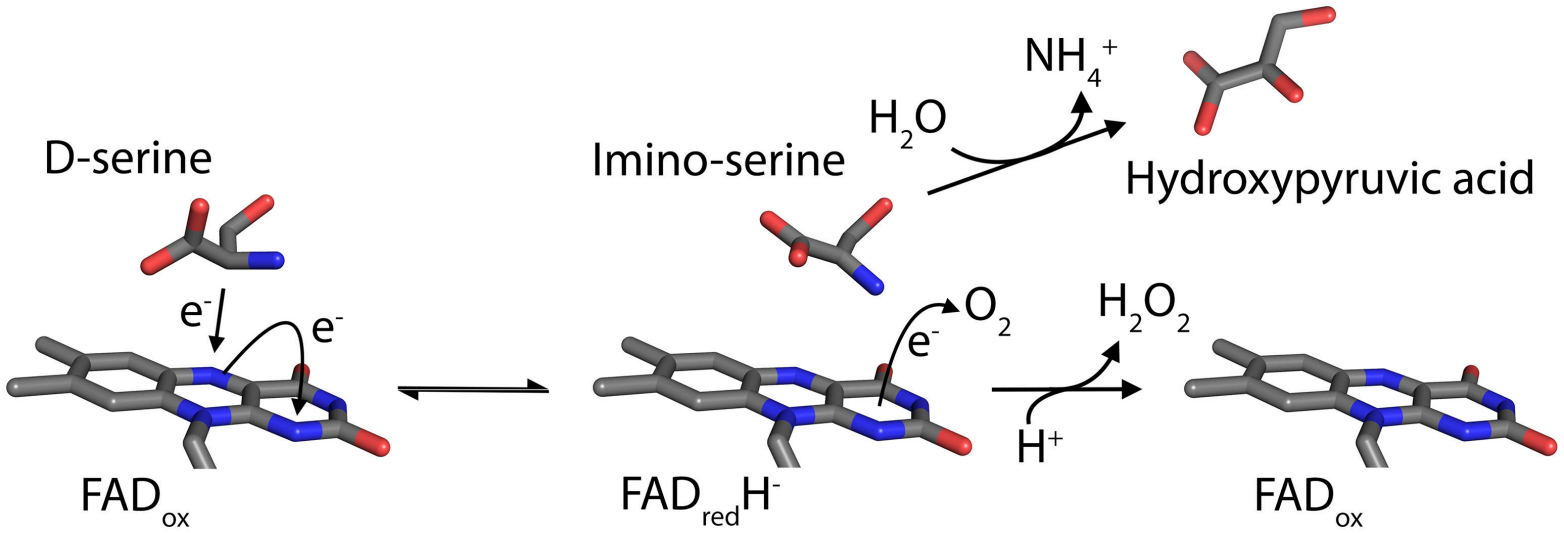
Scheme of the reaction catalyzed by DAAO on the substrate D-Ser.

The peroxisomal flavoprotein D-amino acid oxidase (EC 1.4.3.3, DAAO) is characterized by a strict stereoselectivity and a broad substrate specificity. Since the first identification of the enzyme activity by Krebs in 1935 (Krebs, 1935) and the following purification of the protein from pig kidney (Yagi et al., 1967; Curti et al., 1973), it was subjected to extensive studies becoming the prototype of the dehydrogenase-oxidase class of FAD-dependent flavoenzymes (Fitzpatrick and Massey, 1982). DAAO catalyzes the oxidative deamination of most neutral and polar D-amino acids (D-AAs) to their imino acids counterparts and concomitantly reduces the cofactor FAD. Once released, imino acids non-enzymatically hydrolyze to α-keto acids and ammonia, while the reduced flavin is concomitantly reoxidized by molecular oxygen producing hydrogen peroxide (H_2_O_2_; Figure 1). DAAO is therefore considered a marker enzyme associated with the generation of ROS in peroxisomes.

Despite its atypical substrates, DAAO is widespread in nature: the enzyme activity has been identified in most eukaryotic organisms, the only exception being plants. The protein from a variety of sources, ranging from microrganisms to mammals including humans, have been purified and characterized (Pollegioni et al., 2007; Khoronenkova and Tishkov, 2008). In different organisms, DAAO fulfills distinct physiological functions: it is involved in the catabolism of D-AAs in yeasts (allowing the microorganisms to grow on these substrates as carbon, nitrogen and energy sources); while it plays important regulatory roles in higher organisms. Indeed, in mammals DAAO is implicated in relevant processes ranging from D-AA detoxification and the modulation of excretion in kidney, to neurotransmission in the central nervous system (CNS). Therefore, abnormal levels of the enzyme activity have been shown/proposed to play a significant role in pathologies. Here we provide for the first time a review of human DAAO (hDAAO) variants containing single point amino acidic substitution encoded by single nucleotide polymorphisms (SNPs) reported in the database. Their structural and functional properties, as well as their substantiated or presumed role in diseases, will be discussed.

## DAAO involvement in mammalian key physiological processes

In mammals DAAO is mainly expressed in kidney, liver, brain and to a lesser extent in the small intestine and in neutrophilic leukocytes (Pollegioni et al., 2007, see Table 1). Worthy of note, until 1980s no systematic studies on DAAO from higher organisms were performed for several reasons: the enzyme content and stability were thought to be low, the levels of its substrates D-AAs were barely detectable (their presence in tissues was even questioned) and therefore, no relevant biological functions for the enzyme had been figured out at that time. However, starting from the mid-1990s interest aroused by this enzyme has grown exponentially. Thanks to improved analytical methods based on high performances liquid chromatography (HPLC), significant levels of different D-AAs were detected in brain and other tissues (Nagata et al., 1992; Hashimoto et al., 1993; Hamase et al., 1997). Afterwards, specific physiological roles for D-isomers were demonstrated (Wang et al., 2000; Wolosker et al., 2002; Fuchs et al., 2005) and a key function of DAAO in their metabolic control was proposed (Nagata et al., 1989; Nagata, 1992).

**Table 1:** *DAO* gene and protein expression in humans.

**Tissue**	**Region**	Gene expression^*^ (Log_10_ TPM)	Protein expression^**^	**Cell type**	**Main indicated function(s)**
		** 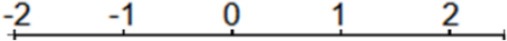 **			
Liver		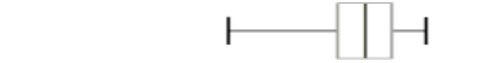	+++	Hepatocytes	Detoxification of DAAs
Kidney	Cortex	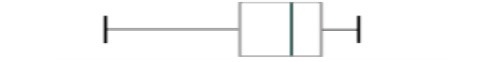	+++	Tubule cells	Detoxification of DAAs, H_2_S generation
Testis		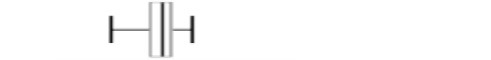	nd		
Colon	Transverse	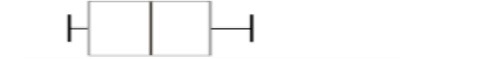	nd		
Pituitary gland		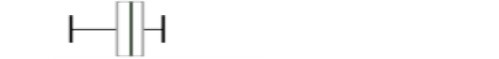	nd		
Spleen		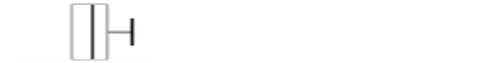	nd		
Whole blood		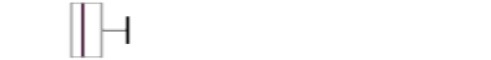	nd		
Small intestine		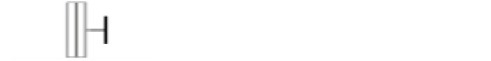	+	Enterocytes	Host defense, controls microbiota composition
Brain	Cerebellum	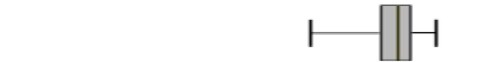	+++	Astrocytes	D-Ser catabolism
	Spinal cord (c-1)	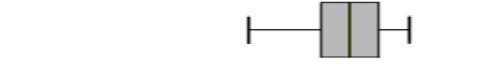	++	Motor neurons	D-Ser catabolism
	Substantia nigra	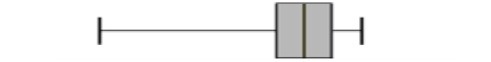	++		D-Ser catabolism, D-DOPA metabolism
	Hypothalamus	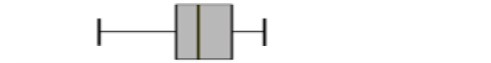	nd		
	Hippocampus	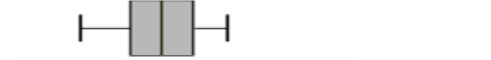	nd		
	Frontal cortex	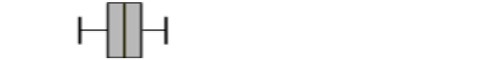	+	Neurons	D-Ser catabolism
	Nucleus accumbens	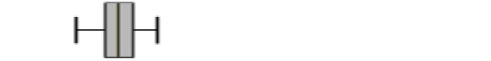	nd		
	Caudate	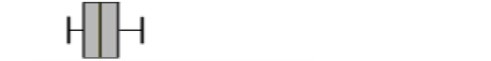	nd		

* *Box plot of DAO gene transcripts levels in different human tissues. Values are shown in TPM (transcripts per kilobase million). Box represent the median and 25 and 75th percentiles. The data presented in this column were obtained from the Genotype-Tissue Expression (GTex) Project Portal, dbGaP accession number phs000424, v7.p2 on 15/02/2018 (https://www.gtexportal.org/home/gene/DAO)*.

** *Expression data as reported on the human protein atlas site (https://www.proteinatlas.org/ENSG00000110887-DAO/tissue) and in the literature cited in this review. + low expression; ++ medium expression, +++ high expression; nd, not detected*.

Liver and kidney are among the most DAAO-rich organs, with the only exception for mouse liver, in which the enzyme has been shown to be absent (Konno et al., 1997). Interestingly, DAAO activity was reported in hepatocytes from mouse fetuses (Dabholkar, 1986), but it disappeared in the liver of adult animals, where DAAO mRNA and protein were undetectable (Konno et al., 1997; Wang and Zhu, 2003). The reason why mouse is the only mammal showing no expression of DAAO in the liver remains an intriguing issue. In kidney, the enzyme was observed in proximal tubule cells by the detection of the encoding transcript (Koibuchi et al., 1995) and enzyme histochemistry in mice (Sasabe et al., 2014). The identification of the mutant ddY/DAAO^−/−^ mice strain, expressing a fully inactive protein variant due to the naturally occurring substitution of a Gly by an Arg at position 181 (Konno and Yasumura, 1983), proved to be of primary importance in the elucidation of DAAO physiological functions. The urine of these animals was reported to contain abnormally high amounts of D-AAs (originating from the cell walls of intestinal bacteria, endogenous racemization or the assumption from the diet). Thus, it was inferred that in liver and kidney (and, to a lesser extent, in the urinary apparatus and in colon) DAAO was responsible for their elimination. More recently, in kidney and brain the enzyme was shown to be involved in the metabolism of the gaseous signaling molecule hydrogen sulfide (H_2_S) through the DAAO/3-MST pathway, an alternative generation pathway (Shibuya et al., 2013).

The flavoenzyme was shown to be also expressed in the granule fraction of normal mature human granulocytes. In these cells DAAO, appropriately linked to myeloperoxidase, was proposed to be part of a cellular strategy to recognize and counteract foreign phagocytized microorganisms (Cline and Lehrer, 1969).

Analogously, the role of DAAO in controlling the homeostasis of gut microbiota has been recently reported (Sasabe et al., 2016). Both enzymatic activity and protein expression were detected in the proximal and middle small intestine of mice and humans (Table 1), associated to the villus epithelium. Unexpectedly, a processed form of the mouse protein appeared to be secreted in the lumen by goblet cells, likely due to the presence of a signal peptide and a predicted cleavage site near the N-terminus (Sasabe et al., 2016). DAAO-mediated oxidative deamination of free D-AAs (present as microbial products) with the consequent production of H_2_O_2_, was shown to represent an important factor in host defense, and in the modulation of microbiota composition (Sasabe et al., 2016).

In the CNS, DAAO was proposed to exert an important physiological function (undoubtedly, the most investigated one). It appeared responsible for the catabolism of endogenous D-Serine (D-Ser), a key neuromodulator for the development of neural circuits, which acts as a co-agonist of N-methyl-D-aspartate receptors (NMDAR). Thus, it was suggested that DAAO contributes to normal neuronal functioning. Consistently, D-Ser levels distribution in the CNS was found to precisely mirror DAAO expression one, both in rodents and humans. The cellular mechanisms involved in the kinetics of serine enantiomers have been the subject of a heated debate. A widely accepted scenario was summarized in the so-called “serine shuttle” model (Wolosker, 2011). According to this hypothesis, D-Ser is predominantly produced in neurons by the stereoconversion from L-serine (provided by astrocytes) catalyzed by the PLP-dependent enzyme serine racemase (SR) (Wolosker, 2011; Wolosker and Radzishevsky, 2013), and shuttled to astrocytes where it is stored and released. Here DAAO, mainly expressed in this cell type contrary to SR, is believed to exert a key role in the regulation of D-Ser cellular concentrations and release, thus indirectly modulating its availability at the synapse and affecting NMDAR activation state through the reduced occupancy of the co-agonist site.

Due to the different methodologies used to detect the presence of DAAO, complexities, and controversies were raised concerning its expression levels and distribution in the mammalian CNS, in terms of both region and cell type (Table 1 summarizes the available data on humans). In rodents, histochemistry methods based on the detection of the enzyme activity led to traditionally consider DAAO as a hindbrain enzyme, highly expressed in the cerebellum (in particular in Bergmann glia), spinal cord and brain stem (Horiike et al., 1994; Kapoor and Kapoor, 1997; Sasabe et al., 2012). On the other hand, immunohistochemistry allowed the detection of low levels of DAAO also in frontal cortex and hippocampus (Moreno et al., 1999).

Both DAAO activity and immunoreactivity was instead consistently detected in the human forebrain, albeit at only a small fraction of that seen in cerebellum (Verrall et al., 2007; Madeira et al., 2008; Table 1). A recent elegant study, performed in both human and mouse tissues, investigated the flavoenzyme distribution using a novel activity staining method based on FITC-conjugated tyramide (Sasabe et al., 2014). The authors confirmed the presence of DAAO in human forebrain regions, but differently from previous studies (Verrall et al., 2007), detected the enzyme activity only in the white matter, throughout the corticospinal tract and in the spinal gray matter, within astrocytes mainly located in the motor pathway. The high hDAAO levels found in spinal cord (Table 1) and brain stem were consistent with its role in preventing excitotoxic cell death in these regions.

Significant levels of hDAAO activity were detected in the nigrostriatal system (Table 1; Sasabe et al., 2014), suggesting that the flavoenzyme might affect not only glutamatergic but also dopaminergic neurons. Intriguingly, hDAAO was shown to efficiently metabolize D-DOPA: it is converted to dihydroxyphenylpyruvic acid, which is then transaminated to L-DOPA via what is known as the alternative pathway for dopamine biosynthesis (Kawazoe et al., 2007a,b; and reference therein). These findings suggested that D-DOPA might be the preferential substrate of hDAAO in the nigrostriatal system instead of D-Ser, and that the enzyme could be implicated in the metabolism of dopamine, norepinephrine, and epinephrine.

Other roles of DAAO, exerted through the degradation of D-Ser or other D-AAs, might be envisaged. Indeed, D-Ser may also modulate glycinergic transmission by antagonizing NR1/NR3A or NR1/NR3B receptors, which are insensitive to glutamate and activated by glycine (Chatterton et al., 2002; Takarada et al., 2009). On the other hand, the alternative DAAO substrate D-proline can activate glycine receptors (Hamasu et al., 2010), whereas D-leucine is a potent regulator of the blood–brain barrier encephalin transport system (Banks and Kastin, 1991). It is still not known, whether these various additional actions of D-AAs may effectively be regulated by DAAO and have any significance with regard to its involvement in pathologies.

## DAAO and pathologies: proposed roles and implications

### Kidney diseases

Experimental evidence pointed out a role of DAAO in some chronic renal pathologic damages, such as D-Ser and D-propargylglycine induced nephrotoxicity (Konno et al., 2000; Maekawa et al., 2005), due to the intracellular DAAO-mediated generation of H_2_O_2_, which in turn can produce even more aggressive ROS (Krug et al., 2007). Accordingly, renal ROS levels exhibits strong dependence on DAAO activity. On the other hand, a more recent study performed in rats reported that renal ischemia-reperfusion injury, a major cause of acute kidney injury, substantially reduced renal DAAO activity (Zhang et al., 2012). The proposed underlying pathophysiological mechanism appeared very complex, involving among others ATP depletion, calcium overload, ROS generation, apoptotic and inflammatory responses (Eltzschig and Eckle, 2011). The observed change in the local level of DAAO activity was attributed to the markedly reduced pH value in kidney upon ischemia (Garcia et al., 2007; Prathapasinghe et al., 2008).

Emerging evidence suggested that H_2_S also actively regulates renal function and is implicated in numerous acute and chronic kidney diseases. The broad renal protective effect of exogenous H_2_S has been recently reviewed (Cao and Bian, 2016), but the systemically administration of H_2_S raised matter of concern due to its well-known toxicity (Guidotti, 2010). Considering the role of DAAO in the DAAO/3-MST H_2_S generation pathway, its targeting by D-cysteine administration has been proposed as a way to safely deliver H_2_S to the kidney (Shibuya et al., 2013).

### Chronic pain and related diseases

Neuropathic pain that arises after nerve injury is characterized by ongoing neurotransmission of pain signals through spinal circuits via the dorsal root ganglion and dorsal horn neurons (Basbaum et al., 2009). NMDARs are expressed in spinal cord neurons and play a key role in the development of ongoing pain states via central sensitization (Latremoliere and Woolf, 2009). A pioneering study reported that tonic pain-related behavior was exaggerated in the ddY/DAAO^−/−^ mice lacking DAAO activity (Wake et al., 2001), indicating that the resulting increased D-Ser levels potentiated NMDARs activation leading to a buildup in the second phase of the formalin response. More recent investigations (Zhao et al., 2010; Gong et al., 2011), further confirmed that DAAO in the spinal cord acts as a pronociceptive factor and demonstrated the efficacy of DAAO inhibitors in models of tonic and chronic pain, generally believed to be mediated by central sensitization. In this context, the systemic administration of the DAAO inhibitor 4H-furo[3,2-b]pyrrole-5-carboxylic acid was shown to reverse pain-related behaviors in rat models of neuropathic and inflammatory pain, with a reduction in the electrophysiological activity in spinal cord dorsal horn neurons and peripheral afferent inputs (Hopkins et al., 2013).

It cannot be excluded that the role of DAAO in chronic pain involves other mechanisms, independent from NMDAR modulation. Indeed, conditions of nerve injury may include changes in local concentrations of ROS, as it has been reported for formalin-induced pain (Lu et al., 2012). The inhibition of DAAO activity was observed to reduce the spinal H_2_O_2_ level (Lu et al., 2012; Gong et al., 2014) and to increase D-Ser levels in the brain and plasma (Adage et al., 2008; Duplantier et al., 2009; Hopkins et al., 2013).

Interestingly, spinal DAAO has been reported to contribute to pain hypersensitivity induced by perturbation of the sleep-regulating circuitries in the CNS (i.e., a “physiological” process) through the deprivation of sleep, that produces pain hypersensitivity without an accompanying nerve or tissue injury (Wei et al., 2013). Also in this case, the role of DAAO has been suggested to be related to the production of ROS species. H_2_O_2_ produced by the enzyme could act on the pronociceptive TRPA1 channel expressed by central terminals of primary afferent nerve fibers in the spinal dorsal horn.

### Neurodegenerative and neuropsychiatric disorders

Multiple lines of evidence suggested that dysfunctions in D-Ser metabolism leading to an excessive or a defective production or release, might be associated with chronic neurodegeneration. D-Ser levels were shown to be greatly increased in the spinal cord of patients with familial and sporadic forms of amyotrophic lateral sclerosis (ALS), altered in Alzheimer's disease affected individuals, and downregulated in schizophrenia (Billard, 2008; Wolosker et al., 2008). Whether the observed abnormal levels depend on the altered expression and/or activity of serine racemase or DAAO is still under investigation.

ALS is the most common adult-onset neuromuscular disorder characterized by the selective degeneration of motor neurons in the spinal cord, brain stem and motor cortex, leading to fatal paralysis. Substantial advances in understanding ALS disease mechanisms has come from the identification of pathogenic mutations in dominantly inherited familial ALS (fALS). Notably, a coding mutation in the *DAO* gene transmitted with the disorder was identified in a three generational fALS kindred (Mitchell et al., 2010). This mutation yielded to a substitution of Arg by Trp at codon 199, and impaired the enzyme activity. The overexpression of R199W hDAAO in primary motor neuron cultures or motor neuron cell lines was reported to promote the formation of ubiquitinated protein aggregates, to activate autophagy and to increase apoptosis, whereas the wild-type protein was without effect on cell survival (Paul and de Belleroche, 2012; Paul et al., 2014). Although these pathogenic effects could be mediated primarily by the accumulation of the mutant protein, other factors might be relevant, such as the impaired activity of DAAO and the consequential dysfunctional effects on D-Ser metabolism. Accordingly, co-culturing motor neurons with glial cells expressing R199W hDAAO variant was sufficient to induce apoptosis in motor neurons, an effect reversed by the treatment with an NMDAR antagonist selective for the D-serine/glycine site. These observations indicated that the way by which the observed neurotoxic effect was transmitted crucially depended on D-Ser (Paul et al., 2014).

Interestingly, studies performed in ddY/DAAO^−/−^ mice showed that these animals developed an abnormal limb reflex and a significant loss of motor neurons in lumbar spinal cord at 8 months (Sasabe et al., 2012). Both in this transgenic line and in the SOD^G93A^ mouse model of ALS, D-Ser was shown to accumulate in the spinal cord during the progression of ALS-related abnormal processes. This was accompanied by a marked suppression of DAAO activity in the reticulospinal tract, a pathway known to play an important role in regulating motor neuron excitability (Sasabe et al., 2012). Consistently to these studies, a recently generated transgenic mouse line expressing R199W DAAO (DAO^R199W^) exhibited the characteristic features of several ALS models including SOD1^G93A^ mice (decreased body weight, marked kyphosis and loss of motor neurons in spinal cord), albeit the overall survival of these animal appeared to be unaffected by the transgene expression (Kondori et al., 2017). Despite no overt ALS phenotype was observed in DAO^R199W^ mice, marked abnormal structural, and motor features associated with a significant loss of lumbar motor neurons, were evident. It was proposed that, as seen in other cases, while a mutation can cause a lethal disease in humans, it may not be possible to generate the same phenotype in a transgenic mouse model carrying the same mutation (Kondori et al., 2017). In fact, hDAAO showed fairly different biochemical properties compared to the rodent's enzyme (Frattini et al., 2011). Notably, enhanced ubiquitination was detected in the cell bodies of large motor neurons of DAO^R199W^ mice, confirming that a prominent feature of the cells expressing the mutant DAAO R199W allele was the presence of ubiquitinated protein aggregates.

Two recent studies have further supported the relevance of DAAO dysfunction to ALS pathogenesis. A comprehensive exome sequencing study revealed that, among the known ALS predisposition gene, *DAO* is the only one where the presence of DNA variants is significantly associated with clinical outcome, decreasing rates of survival (Cirulli et al., 2015). In a work investigating the function of an RNA binding protein encoded by the *HnRNPA2B1* gene, and the effect of the ALS-associated mutation D290V (promoting the protein aggregation in the nucleus and abnormal splicing events), it was found that the most significant and robust splicing change after depletion of hnRNP A2/B1 in the mouse spinal cord was the skipping of exon 9 within *DAO* gene, yielding to a shorter transcript isoform (Martinez et al., 2016). This alteration resulted in a reading frameshift and early termination of the protein. The resulting DAAO isoform was predicted to lack 2 α-helices and 3 β-sheets: it was highly unstable and its enzymatic activity was largely impaired.

Beside motor disorders, Alzheimer's disease (AD) is characterized by cognitive impairments such as dementia and neurodegeneration. Although different mechanisms, such as neuronal apoptosis and inflammatory responses, are thought to be involved in the pathogenesis, increasing evidence has suggested that alterations in various receptors might account for the progression of cognitive decline. Excitotoxicity (i.e., the cell death mediated by calcium overload) induced by the overstimulation of NMDAR has been indicated as a potent mechanism in AD pathophysiology (Tannenberg et al., 2004). In this regard, elevated D-Ser levels were reported in patients (Madeira et al., 2008) and could be considered as a pro-death signal in AD that promotes, in conjunction with glutamate, the neurotoxicity exhibited by inflammatory processes. However, the role of NMDAR activity in AD appeared to be more complex. Different studies showed impaired NMDAR signaling pathway in the cerebral cortex and hippocampus of aging brains (Billard, 2008): a defective activation of NMDAR due to reduced D-serine level was shown to occur in aged tissues (Junjaud et al., 2006; Mothet et al., 2006). Being synaptic NMDAR-mediated transmission crucial to neuronal survival, the impaired activation of the receptor (inducing apoptosis and neuronal cell death) might result in loss of neuronal plasticity and cognitive deficits in the aging brain (Ikonomidou et al., 1999; Lei et al., 2008). This NMDAR hypoactivity-induced neurodegeneration was postulated to contribute to AD pathogenesis (Olney et al., 1997; Wozniak et al., 1998) and it could be involved in the progression of aging brain from mild cognitive impairment to AD. In this regard, it has been proposed that NMDAR-enhancing agents might be beneficial for the early declining process of AD. Supporting this idea, D-Ser was shown to exert a neuroprotective effect against apoptosis, promoting neuronal survival (Esposito et al., 2012) and the DAAO competitive inhibitor sodium benzoate was found to ameliorate cognitive and overall function in patients with early phase AD (Lin et al., 2014). Furthermore, recently increased levels of DAAO have been reported in the serum of mild cognitive impairment patients, as well as mild and severe AD patients, compared to healthy individual (Lin et al., 2017). Worthy of note, the flavoenzyme levels increased with the severity of cognitive deficit and were significantly associated with D-Ser serum content.

Recent studies highlighted the role of synaptic transmitters and their receptors in the etiology of psychiatric disorders. Converging pharmacological, genetic, and neuropathological studies have led to the widely accepted NMDAR hypofunction model of schizophrenia (Coyle et al., 2003; Coyle, 2006; Stone and Pilowsky, 2007). In particular, a deficiency of D-Ser signaling was proposed to be responsible for the altered activation state of the receptor. Several lines of evidence supported this hypothesis: (i) decreased D-Ser levels were reported in serum and cerebrospinal fluid of schizophrenia affected individuals (Hashimoto et al., 2003, 2005); (ii) clinical trials demonstrated the beneficial effects of D-Ser as an add-on therapy to antipsychotic treatment (Heresco-Levy et al., 2005); (iii) D-Ser produced behavioral and neurochemical alterations consistent with clinical effects when administered to animal models (Verrall et al., 2010 and references therein). These findings suggested that DAAO, through its role in D-Ser metabolism might contribute to the proposed NMDAR dysfunction in schizophrenia. This hypothesis was strengthened by the discovery of its interaction with the product of the primate specific gene *G72*, linked to schizophrenia and encoding the small protein pLG72. Indeed, it was shown to modulate DAAO activity, even though along the years the effect of the DAAO-pLG72 protein complex formation has been contrasting: at first, pLG72 was proposed to act by increasing the enzyme activity (and accordingly it was defined DAAO activator, DAOA; Chumakov et al., 2002); while subsequent *in vitro* and cellular studies repeatedly and consistently indicated that pLG72 negatively affected DAAO functional properties, and acted by reducing its activity and destabilizing the protein structure (Sacchi et al., 2008, 2016; Pollegioni et al., 2018). Although some aspects of DAAO-pLG72 interaction need further investigations, several meta-analyses have provided a moderate degree of support for a genetic association between *G72, DAO*, and schizophrenia, thus sketching them in the category of schizophrenia susceptibility genes.

DAAO involvement in the pathophysiology of the neuropsychiatric disease was proposed also based on altered levels of enzyme expression and activity detected in post-mortem brain tissues from affected individuals, rather than merely on genetic analysis, which can be inconclusive in the case of a complex multifactorial disorder. Increased enzymatic activity and gene or protein expression were reported in cerebral cortex (Madeira et al., 2008), cerebellum (Kapoor et al., 2006; Verrall et al., 2007; Burnet et al., 2008), medulla oblongata and choroid plexus (Ono et al., 2009) of schizophrenic patients, suggesting that the onset of the disease is associated with abnormal DAAO levels in different brain areas.

### hDAAO biochemical properties

**Figure 2:**
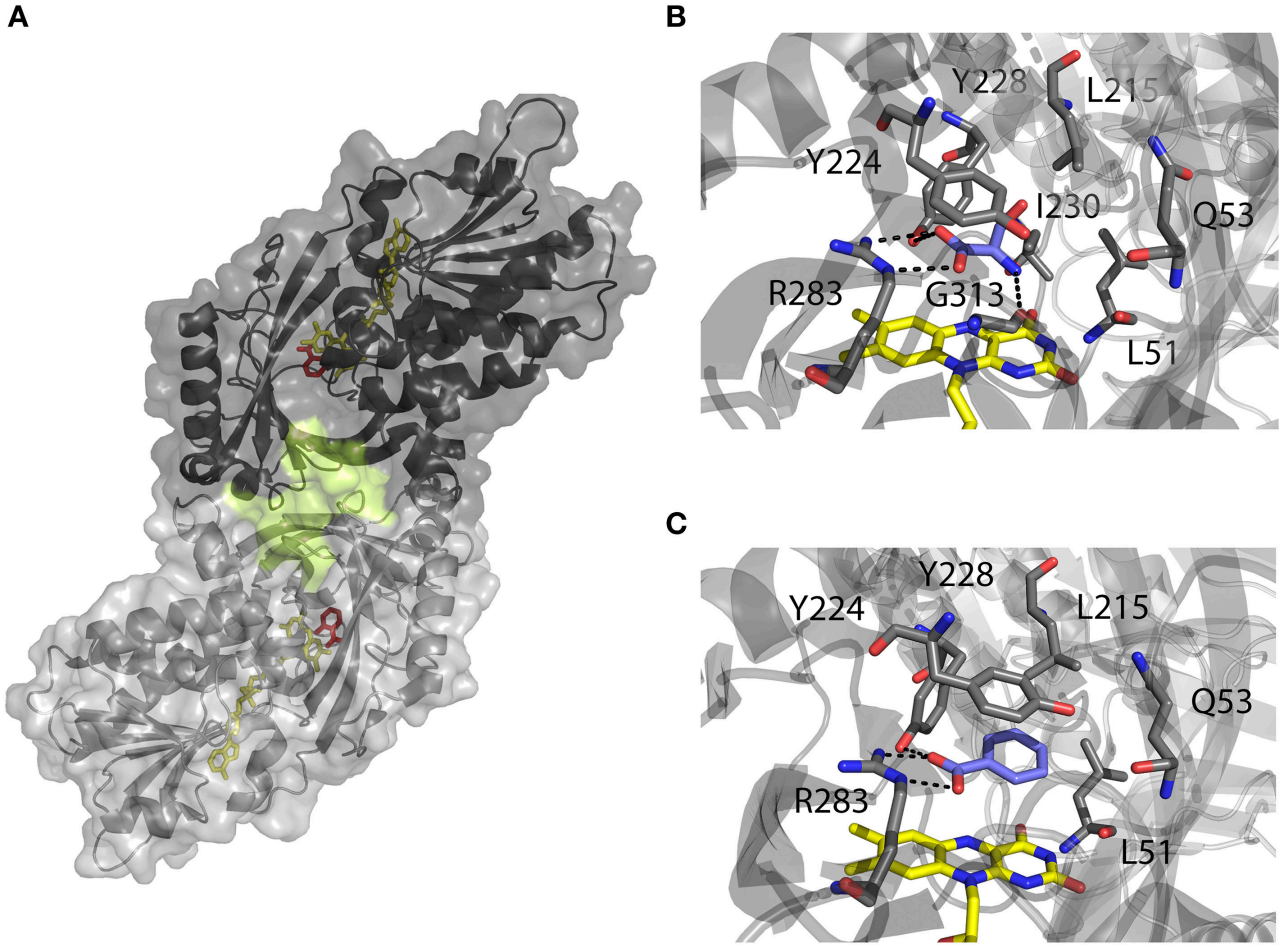
Three-dimensional structure of hDAAO holoenzyme (PDB code 2DU8; Kawazoe et al., 2006). **(A)** Schematic view of the head-to-head mode of monomers interaction. α-helices, β-strands and coils are drawn in cartoon representation. The FAD cofactor and the inhibitor sodium benzoate are shown as sticks, colored in yellow and red, respectively. The protein surface is drawn in dark and light gray, and the second putative ligand binding pocket located at protein monomer interface, is highlighted. **(B, C)** Details of the active site of the enzyme in complex with **(B)** imino-serine (PDB 2E49; Kawazoe et al., 2007a), and **(C)** benzoate (PDB 2DU8; Kawazoe et al., 2006). FAD (yellow), iminoserine and benzoate (blue) as well as side chains of important residue in the active are shown as sticks. Figures were prepared using PyMol.

hDAAO structural and biochemical properties have been extensively investigated using the recombinant enzyme produced in *E. coli* (Molla et al., 2006; Sacchi et al., 2008; Caldinelli et al., 2010). Meanwhile, the crystallographic structure was resolved (Kawazoe et al., 2006). The human enzyme was reported to be a stable homodimer, characterized by a head-to-head mode of monomer interaction (Figure 2A). Each monomer (347 amino acids, 40.3 kDa) was shown to contain a non-covalently bound FAD molecule and to be composed by 11 α-helices and 14 β-strands, that fold into two interconnected regions: a substrate binding domain, with a large twisted antiparallel β-sheet forming the active-site roof and part of the oligomerization interface; and a FAD binding domain containing the dinucleotide binding motif (Rossmann fold). The cofactor was found to be buried in the protein core in an elongated conformation, with the isoallxazine ring located at the interface of the two domains and the *re*-face in the inner part of the active site (Kawazoe et al., 2006; Molla, 2017).

Based on the enzyme three-dimensional structure, several considerations can be made. The substrate binds to the active site where it is positioned above the *re*-side of the cofactor, in the correct orientation with respect to the reactive N(5) of the isoalloxazine moiety: the dehydrogenation of the substrate occurs by the direct hydride transfer of the α-hydrogen from the α-carbon of the D-amino acid to the flavin N(5) (Figure 1). Several H-bond interactions concur in fixing and stabilizing the substrate in the active site (Figure 2B). Namely, α-COOH group of the substrate interacts with the active site residues Arg283 and Tyr228, whereas the α-NH_2_ group is connected with Gly313 and the C(4) = O of the cofactor. The substrate side chain is located in an active site pocket made up by bulky and hydrophobic residues (i.e., Leu51, Gln53, Leu215, and Ile230; Kawazoe et al., 2006). Further, the “roof” of the active site is formed by the side chain of Tyr224 that is part of a mobile loop (216-228), which switches from a close to an open conformation to allow the product/substrate exchange during the catalysis.

hDAAO showed peculiar biochemical properties. Differently to others DAAOs (Mattevi et al., 1996; Pollegioni et al., 2007; Frattini et al., 2011), the enzyme appeared to be a homodimer, in both the holo- and the apoprotein form (Molla et al., 2006). This was proposed to depend on a distinctive charge distribution at the dimer interface, where a significantly higher amino acidic substitution frequency compared to the overall protein was observed (33% vs. 15%, respectively; Kawazoe et al., 2006). The binding of the FAD cofactor to the human enzyme was determined as the weakest among known DAAOs (K_d_ = 8.0 μM for hDAAO vs. 0.2 and 0.02 μM for pig kidney and yeast DAAOs, respectively), although the presence of a ligand in the active site stabilized the flavin interaction (K_d_ = 0.3 μM) (Molla et al., 2006; Caldinelli et al., 2010). Due to the low affinity of FAD interaction, hDAAO was reported to be present in solution as an equilibrium of holo- and apoprotein forms (Caldinelli et al., 2010). Benzoate binding has been recently shown to promote a conformational switch yielding a form with a higher avidity for FAD (Murtas et al., 2017a). DAAO-catalyzed oxidative deamination was demonstrated to follow a ternary-complex mechanism (Pollegioni et al., 1993; Umhau et al., 2000). For hDAAO the reductive half-reaction was very fast (117 ± 6 s^−1^ on D-Ser) but the turnover was much slower (6.3 ± 1.4 s^−1^), the product release representing the rate-limiting step (Molla et al., 2006; Molla, 2017). Because of the plasticity of the active site lid, hDAAO showed a wide substrate specificity. Best substrates were shown to be hydrophobic D-AAs (D-DOPA > D-Tyr > D-Phe > D-Trp); among them D-DOPA yielded to the highest *k*_*cat*_ value, although different kinetic parameters were reported (Kawazoe et al., 2007a; Murtas et al., 2017a). The human enzyme was also reported to catabolize small uncharged D-AAs (D-Cys > D-Ala > D-Pro > D-Ser) (Molla et al., 2006; Kawazoe et al., 2007a; Frattini et al., 2011; Murtas et al., 2017a). Notably, some of them play a relevant role in neurotransmission. In particular, hDAAO showed the highest catalytic efficiency for D-cysteine, an intermediate of H_2_S metabolic pathway (see above; Murtas et al., 2017a). Glycine and acidic D-AAs were only poorly degraded by the human enzyme (Molla et al., 2006; Murtas et al., 2017a).

It has been demonstrated that hDAAO functionality is modulated by the interaction with other proteins. In particular, the enzyme was shown to specifically bind to the small and primate-specific protein pLG72: two hDAAO homodimers interacted with two pLG72 molecules in a 200 kDa protein complex (for a recent review on pLG72-hDAAO interaction see Pollegioni et al., 2018). *In vitro*, the complex formation modified hDAAO tertiary structure, causing a time-dependent loss of activity (Sacchi et al., 2008). Accordingly, at the cellular level hDAAO-pLG72 interaction positively affected D-Ser cellular levels and decreaseed hDAAO half-life (Sacchi et al., 2008, 2011; Cappelletti et al., 2014). The structural details of the interaction remain to be fully elucidated: a prominent role of the C-terminal region in the modulation of hDAAO activity was initially proposed (Chang et al., 2013); but further analyses performed by using different recombinant pLG72 deletion variants, while confirming that C-terminal terminal region is likely required to induce the alterations in hDAAO conformation associated with the loss of catalytic functions, indicated that the N-terminal region of the protein was crucial for the strength of the protein complex formation (Birolo et al., 2016). The enzyme activity was reported to be also negatively modulated by bassoon, a protein of the cytoskeletal matrix, enriched in the presynaptic active zone. hDAAO-bassoon interaction was proposed to play a homeostatic role in preventing D-Ser depletion by an active and extraperoxisomal hDAAO form detected in at presynaptic terminals (Popiolek et al., 2011). Worthy of note, bassoon inhibitory effect might in part explain why hDAAO enzymatic activity was barely detectable in the forebrain (Verrall et al., 2007), where hDAAO was thought to be mainly expressed in neurons, albeit at fairly low levels.

Since abnormal changes in hDAAO activity leading to locally decreased D-Ser levels have been correlated with severe neurological disorders (such as schizophrenia), the design, and selection of hDAAO inhibitors to be used as drugs has gained a growing interest (Ferraris et al., 2008). The mode of binding of the substrate-competitive inhibitor benzoate to the hDAAO active site is shown in Figure 2C. An exhaustive dissertation about the structural details relevant for hDAAO inhibitors binding has been recently published (Molla, 2017).

Despite the tremendous work that has been done so far, there are still some aspects concerning hDAAO structural/functional relationships that must be clarified: recent computational and labeling analyses has suggested the presence of an unexpected, additional ligand binding site located at the interface between the two monomers of the holoenzyme (Figure 2A; Kohiki et al., 2017). This hypothesis was supported by experimental evidences showing two different phases in benzoate binding to the holoenzyme, and indicating the existence of two different conformations with different ligand affinities or two different ligand binding sites (Murtas et al., 2017a).

Noteworthy, altogether these investigations suggest that evolution has adopted sophisticated strategies to finely modulate the activity of hDAAO in order to use this enzyme in different tissues responding to several needs.

## Inactive hDAAO variants potentially related to neurodegenerative disorders

**Figure 3:**
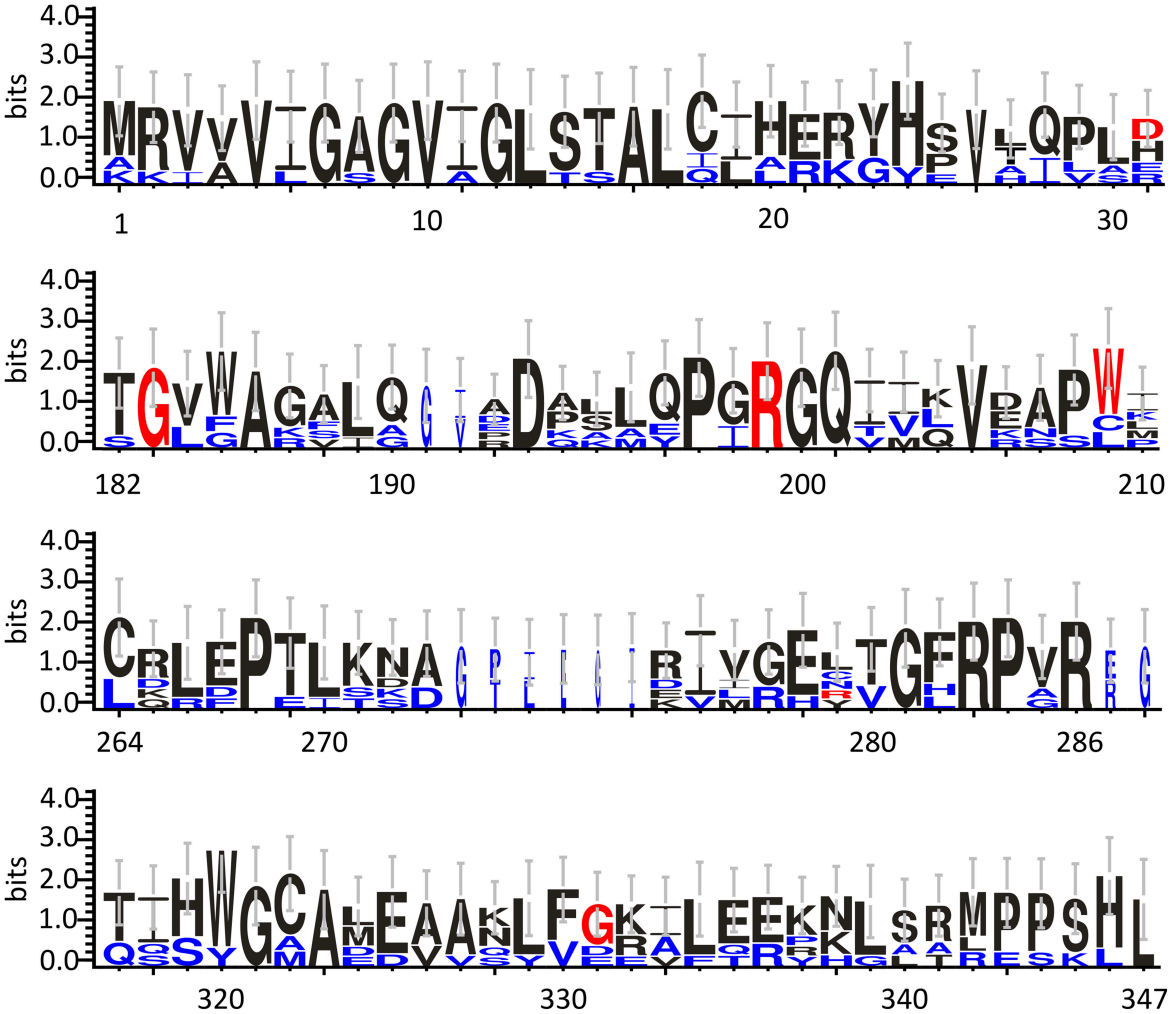
Frequency of sequence conservation among DAAOs from different sources. WebLogo representation of conserved residues identified by the alignment of DAAOs from mammals (*Homo sapiens, Mus musculus, Rattus norvegicus, Sus scrofa*) and yeast (*Rhodotorula gracilis* and *Trigonopsis variabilis*). The x-axis represents amino acid position (the annotated numbering refers to the human enzyme). The y-axis indicates the sequence conservation at that position (measured in bits), whereas the height of symbols is proportional to degree of conservations of single residues. Panels represent sequence stretches of 31 amino acids containing the residues that are substituted in the hDAAO variants discussed in this review (shown in red). Residues belonging only to the yeast sequences are shown in blue. Figure prepared using WebLogo 3.0 (Crooks et al., 2004).

Based on cellular and biochemical studies, the R199W, R199Q, G183R, and G331V hDAAO variants could be related to the onset of neurodegenerative diseases. For all these protein variants, the substitution significantly reduced (when not fully abolished) the enzyme activity. This in turn, might result in increased D-Ser levels at the synapses and a consequent hyperactivation of NMDAR, a condition known to induce cytotoxicity and neuronal cell death (Mitchell et al., 2010). The G183R hDAAO variant corresponds to the coding SNP that naturally occurs in the ddY/DAAO^−/−^ mice strain expressing the homologous G181R DAAO characterized by the complete loss of the enzymatic activity (Konno and Yasumura, 1983). On the other hand, the R199W hDAAO variant has been associated with the onset of fALS (Mitchell et al., 2010) and, to date, it is the unique hDAAO variant linked to a human disease. The R199W substitution was shown, not only to strongly affect the enzyme activity (Cappelletti et al., 2015), but also to promote protein aggregation and to be responsible of primary motor neuron degeneration (Mitchell et al., 2010; Paul and de Belleroche, 2012). The R199Q hDAAO variant corresponds to a confirmed substitution reported in the SNPs database (rs200850756). As expected, this protein variant showed biochemical features resembling those reported for the R199W one (Cappelletti et al., 2015). Finally, the G331V substitution is encoded by the reported hDAAO SNP rs4262766. Its overexpression in U87 human glioblastoma cell line resulted in low viability of the transfected cells, since it was shown to promote the formation of toxic protein aggregates and to induce apoptosis (Caldinelli et al., 2013). Among the aforementioned substituted residues, G183 and R199 are highly conserved in DAAOs from different sources (Figure 3).

**Figure 4:**
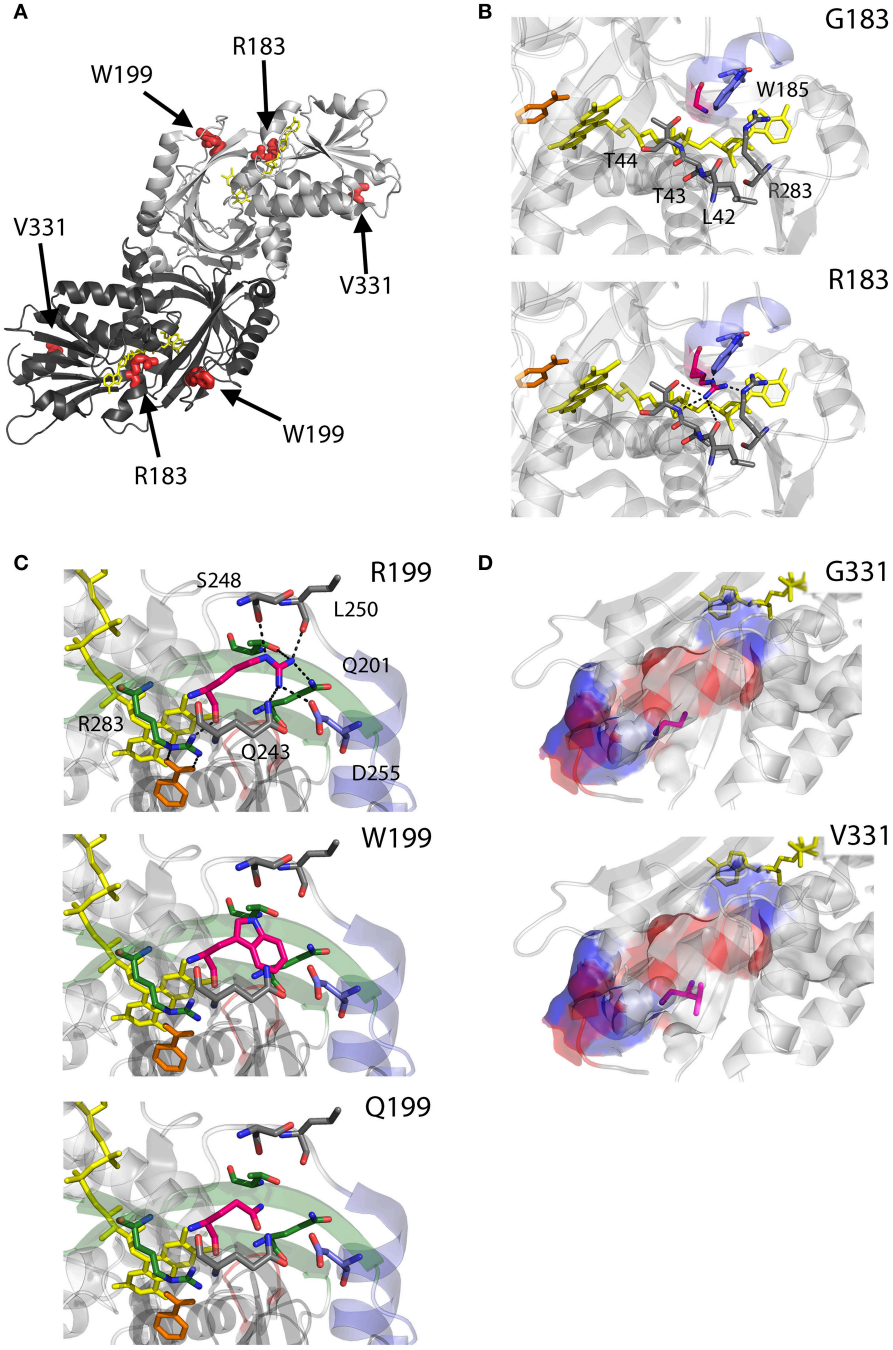
Structural models of hDAAO inactive variants. **(A)** The position of the point mutations on the protein dimeric structure (PDB code: 2DU8; Kawazoe et al., 2006) is shown. Substituted residues encoded by SNPs are depicted as thick sticks in red, while FAD cofactor is reported in yellow. **(B–D)** Details of the protein microenvironment surrounding at the mutated residues in the different hDAAO variants. The substituted residues are shown as sticks in magenta. The surrounding residues within a distance of 4 Å are shown as sticks in gray. Loops, α-helices and β-sheets indicated in the text are shown as cartoon in red, blue and green, respectively. The substrate-competitive inhibitor benzoate and the FAD cofactor are shown as sticks in orange and yellow, respectively. H-bond interactions are reported as black dashed line. Negatively charged (blue), positively charged (red) and neutral (gray) surfaces of the C-terminal α-helix are shown in **(D)**. Figure were prepared with PyMol. Adapted from Caldinelli et al. (2013); Cappelletti et al. (2015); Murtas et al. (2017b).

As we can deduce from hDAAO three-dimensional structure, G183 belongs to the α9 α-helix, which is close to pyrophosphate and ribityl groups of the cofactor (Figures 4A,B). Here, the substitution of the wild-type residue with an arginine likely leads to additional electrostatic and H-bond interactions with the surrounding residues side chain, as well as with the –CO and –NH backbone groups that are in close contact with the adenine nucleotide moiety of FAD (Figure 4B). It has been proposed that this results in local alterations of the protein variant conformation that negatively affect the cofactor binding and its correct orientation required for hydride transfer during catalysis (Murtas et al., 2017b).

R199 is on the β-strand β8, which lies close to the FAD and substrate binding site. Intriguingly, this residue is placed at the protein surface (~11 Å from the isoalloxazine ring, thus pretty close to the active site), at the center of a tight H-bond network with residues of α-helix α10 and β-strands β8 and β12, secondary structure elements belonging to the interface domain (Figures 4A,C). Moreover, since R199 interacts with R283, its substitution might affect the conformation of this key active site residue, possibly impairing the ability of hDAAO variants to stabilize the binding of substrate/inhibitors. Indeed, not only the carbonyl group on the main chain of R199 directly interacts with R283 side chain, but the side chain is H-bonded to T280 belonging to the same β-strand of R283 (β12) (Figure 4C; Cappelletti et al., 2015). In this context, the substitution of R199 with a Trp has been proposed to be responsible of a significant alteration in the conformation of the loop between β11 and α10 due to the different steric hindrance. On the other hand, the substitution with a Gln likely causes the loss of a positive charge and consequently of the existing electrostatic interaction. Altogether, the R199W and R199Q have been suggested to promote the disruption of relevant interactions, thus affecting the overall protein conformation and the enzymatic activity (Cappelletti et al., 2015).

On the other hand, G331 appears to be highly conserved in higher organisms only (Figure 3). It is located on the C-terminal α-helix, near the protein surface, apparently exposed to the solvent (Figures 4A,D). G331 is far from both the active site and the interface between monomers, in a region probably involved in the protein folding process and exposed in the folding intermediate(s) (Caldinelli et al., 2004). In this case, the substitution of the wild-type Gly with a Val has been proposed to affect the helix structure and produce a hydrophobic surface, which makes the hDAAO variant prone to aggregation (Caldinelli et al., 2013).

### Expression in *E. coli* and biochemical properties

The different hDAAO variants have been overexpressed in *E. coli* as recombinant proteins fused with an N-terminal 6XHis-Tag, and subjected to further characterization. Growth conditions were modified and adapted for each hDAAO variant (Caldinelli et al., 2013; Cappelletti et al., 2015; Murtas et al., 2017b), since using the expression conditions set up for the wild-type enzyme all the protein variants were largely produced as inclusion bodies, in the insoluble fraction of the cell extract. Despite the optimized fermentation protocols led to increased levels of soluble protein expression, the purification yield was always lower than for the wild-type protein (see Table 2). The G183R hDAAO was purified as fully inactive apoprotein (Murtas et al., 2017b). Analogously, the purified R199W and R199Q variants were largely purified in apoprotein form, and showed a significantly reduced specific activity compared to the wild-type hDAAO (Table 2; Cappelletti et al., 2015). On the other hand, the expression and purification yields for the G331V hDAAO were consistently too low to allow a detailed biochemical characterization. However, its estimated specific activity closely resembled the value determined for the recombinant wild-type hDAAO (Table 2; Caldinelli et al., 2013).

**Table 2:** Expression conditions of recombinant hDAAO variants in *E. coli*.

**hDAAO variant**	**Wild-type^a^**	**G183R^b^**	**R199Q^c^**	**R199W^c^**	**G331V^d^**	**D31H^d^**	**W209R^c^**	**R279A^d^**
*E. coli* strain	BL21	BL21	BL21	BL21	Origami	BL21	BL21	BL21
	(DE3)	(DE3)	(DE3)	(DE3)	(DE3)	(DE3)	(DE3)	(DE3)
	STAR	STAR	STAR	STAR		STAR	STAR	STAR
**FERMENTATION CONDITIONS**								
Cultivation broth	TB	TB	TB	TB	TB	TB	TB	TB
Riboflavin addition (μM)	/	/	/	10	/	/	/	/
IPTG (mM)	0.1	0.6	0.6	0.6	0.1	0.1	0.6	0.1
Temperature after induction (°C)	37	37	30	23	37	37	37	37
Time of collection after induction (h)	4	20	20	2	4	4	20	4
Purified protein (mg/L)	7.0	5.4	2.0	3.0	0.1	20.0	10.0	10.0
Specific activity (U/mg)	12.0	/	1.2	4.1	2.9 (11.6)^*^	14.0	12.0	10.5
Purity (%)	≥90	≥90	≥90	≥90	25	≥90	≥90	≥90

*The specific activity was determined using the oxygen consumption assay (0.25 mM oxygen, 25°C) at 28 mM D-alanine in presence of an excess of FAD (200 μM)*.

* *Value estimated based on the ~ 25% purity (see text for details)*.

a *Molla et al., 2006*;

b *Murtas et al., 2017b*;

c *Cappelletti et al., 2015*;

d *Caldinelli et al., 2013*.

Several attempts were performed to reconstitute G183R hDAAO variant (Murtas et al., 2017b); however, the resulting preparation, despite showing the absorbance spectrum of the oxidized holoenzyme, was still inactive. R199W and R199Q hDAAO variants showed a dramatically reduced catalytic efficiency with respect to the wild-type enzyme, mostly due to a consistent increase in the K_m_ values (Table 3). Notably, when measurements were performed using 1 mM D-Ser (a concentration similar to that detected in normal brain; Hashimoto et al., 1995) the activity of R199W and R199Q hDAAO variants was undetectable (Cappelletti et al., 2015).

**Table 3:** Apparent kinetic parameters of hDAAO variants.

**hDAAO variant**	**D-Serine**			**D-Alanine**		
	**k_cat_ (s^−1^)**	**K_m_ (mM)**	**k_cat_/K_m_ (s^−1^m M^−1^)**	**k_cat_ (s^−1^)**	**K_m_ (mM)**	**k_cat_/K_m_ (s^−1^mM^−1^)**
Wild-type^a^	3.0 ± 0.1	7.5 ± 3.1	0.4	5.2 ± 0.2	1.1 ± 0.2	4.7
R199Q^b^	≥8	≥2000	0.004	≥10	≥2000	0.005
R199W^b^	≥15	≥2000	0.0075	~15	~ 400	0.04
D31H^c^	3.0 ± 0.2	3.9 ± 0.9	0.77	6.0 ± 0.2	0.95 ± 0.1	6.3
W209R^b^	7.2 ± 0.2	17.3 ± 3.5	0.42	11.8 ± 0.2	1.2 ± 0.2	8.6
R279A^c^	2.5 ± 0.1	3.4 ± 0.5	0.74	8.6 ± 0.1	1.0 ± 0.1	8.6

*The activity was assayed at air saturation (0.25 mM oxygen), 25°C, and pH 8.5*.

a *Molla et al., 2006*;

b *Cappelletti et al., 2015*;

c *Caldinelli et al., 2013*.

Furthermore, a significantly decreased cofactor binding affinity was observed for the two hDAAO variants, both in the free and benzoate complexed forms (Table 4; Cappelletti et al., 2015). On the other hand, the G183R hDAAO variant retained the FAD binding affinity, albeit the strength of the interaction drastically decreased in presence of an excess of benzoate (Table 4; Murtas et al., 2017b). The binding properties of all the variants in general appeared altered: an overall decrease in binding affinity for known competitive inhibitors compared to the wild-type enzyme has been observed (Table 4). Notably, the most pronounced changes were detected for the R199W and R199Q variants; in particular, the estimated K_d_ values for benzoate were 300-fold and 600-fold higher compared to the wild-type one, respectively. The ability of R199Q and G183R hDAAO to interact with the regulatory protein pLG72 was unaffected by the substitution (Cappelletti et al., 2015; Murtas et al., 2017b; Table 4). In the case of the R199W hDAAO instead, its oligomeric state (see below) made unfeasible the identification of the hDAAO-pLG72 protein complex formation.

**Table 4:** Binding properties of hDAAO variants.

**hDAAO variant**	**FAD (K**_**d**_, μ**M)^a^**		**Benzoate (K_d_, μM)**	**CBIO (K_d_, μM)**	**CPZ^a^ (K_d_, μM)**	**pLG72^c^ interaction**
	**Free form**	**Benzoate complex**				
Wild-type^d^	7.9 ± 0.2	0.3 ± 0.1	7.0 ± 2.0^b^	101 ± 24.0^b^	5.0 ± 0.1	Yes
G183R^e^	8.4 ± 1.9	235 ± 26.0	6500 ± 400^a^	628 ± 25.0^a^	9.3 ± 0.2	Yes
R199Q^f^	33.5 ± 1.6	28.4 ± 3.9	4160 ± 230^b^	342 ± 11.0^b^	16.2 ± 5.3	Yes
R199W^f^	40.3 ± 2.6	14.6 ± 1.4	2140 ± 130^b^	157 ± 30.0^b^	11.1 ± 1.6	n.d.
D31H^g^	1.8 ± 0.1	0.13 ± 0.02	4.6 ± 0.4^b^	n.d.	4.8 ± 0.3	Yes
W209R^f^	1.9 ± 0.1	0.15 ± 0.001	3.9 ± 1.2^b^	96.0 ± 36.0^b^	2.0 ± 0.8	Yes
R279A^g^	0.9 ± 0.1	0.03 ± 0.002	8.0 ± 0.5^b^	n.d.	6.4 ± 0.3	Yes

a *K_d_ values determined by monitoring the quenching of protein fluorescence*.

b *K_d_ values determined by monitoring the perturbation of flavin absorbance spectra*.

c *Analysis of pLG72 binding performed by gel-permeation chromatography. The hDAAO R199W elution volume is close to that of the hDAAO-pLG72 complex, making unfeasible the identification of the complex*.

*n.d. = not determined*.

d *Molla et al., 2006*;

e *Murtas et al., 2017b*;

f *Cappelletti et al., 2015*;

g *Caldinelli et al., 2013*.

### Alterations in protein conformation

**Figure 5:**
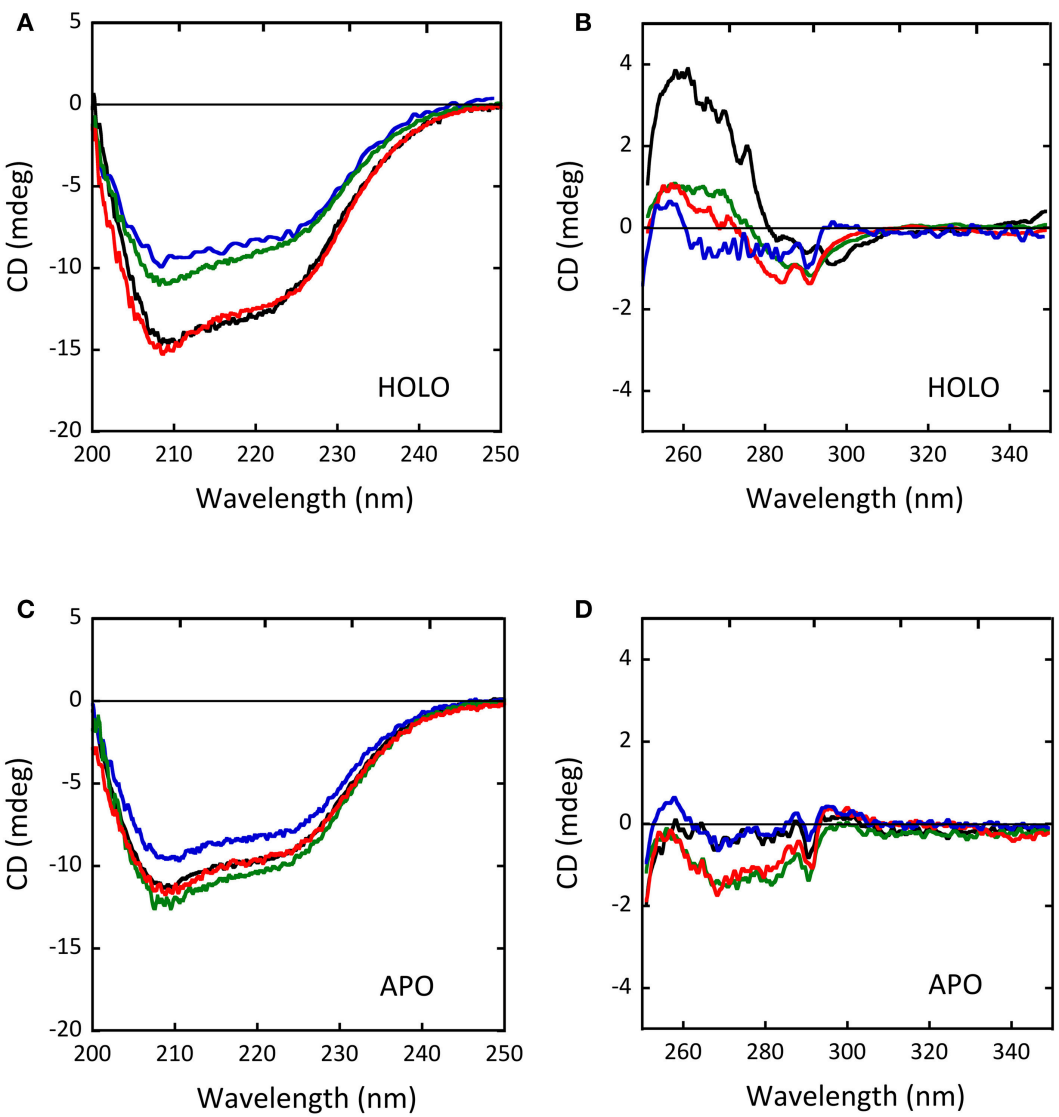
Comparison of the CD spectra of wild-type (black), G183R (blue), R199W (green) and R199Q (red) hDAAO variants. **(A,C)** Far-UV CD spectra of **(A)** the holoenzyme and **(C)** the apoprotein forms of the hDAAO variants (0.1 mg/mL protein concentration). **(B,D)** Near-UV CD spectra of **(B)** the holoenzyme and **(D)** the apoprotein forms of the hDAAO variants (0.4 mg/mL protein concentration). From (Caldinelli et al., 2013; Cappelletti et al., 2015; Murtas et al., 2017b).

As determined by circular dichroism (CD) analyses, the G183R, R199W, and R199Q hDAAO holenzymes conformation appeared to be modified with respect to the wild-type holoenzyme (Figure 5): while minor changes in secondary structure elements content were observed for G183R and R199W protein variants (Figure 5A), their tertiary structure appeared to be specially affected (Figure 5B). In particular, the near-UV CD spectrum of the G183R hDAAO holoenzyme strictly resembled the apoprotein one. Only minor alterations compared to the wild-type enzyme were instead detected in the near-UV CD spectra of the apoprotein forms of the different hDAAO variants (Figures 5C,D; Cappelletti et al., 2015; Murtas et al., 2017b). Furthermore, protein stability studies performed by analyzing the temperature sensitivity of parameters that allowed to monitor changes in secondary and tertiary structures (the CD signal at 220 nm and tryptophan fluorescence, respectively), revealed that the melting temperature estimated for the R199W and R199Q variants was up to 13°C higher compared to the wild type one (Cappelletti et al., 2015), pointing to a decrease in the protein flexibility.

**Figure 6:**
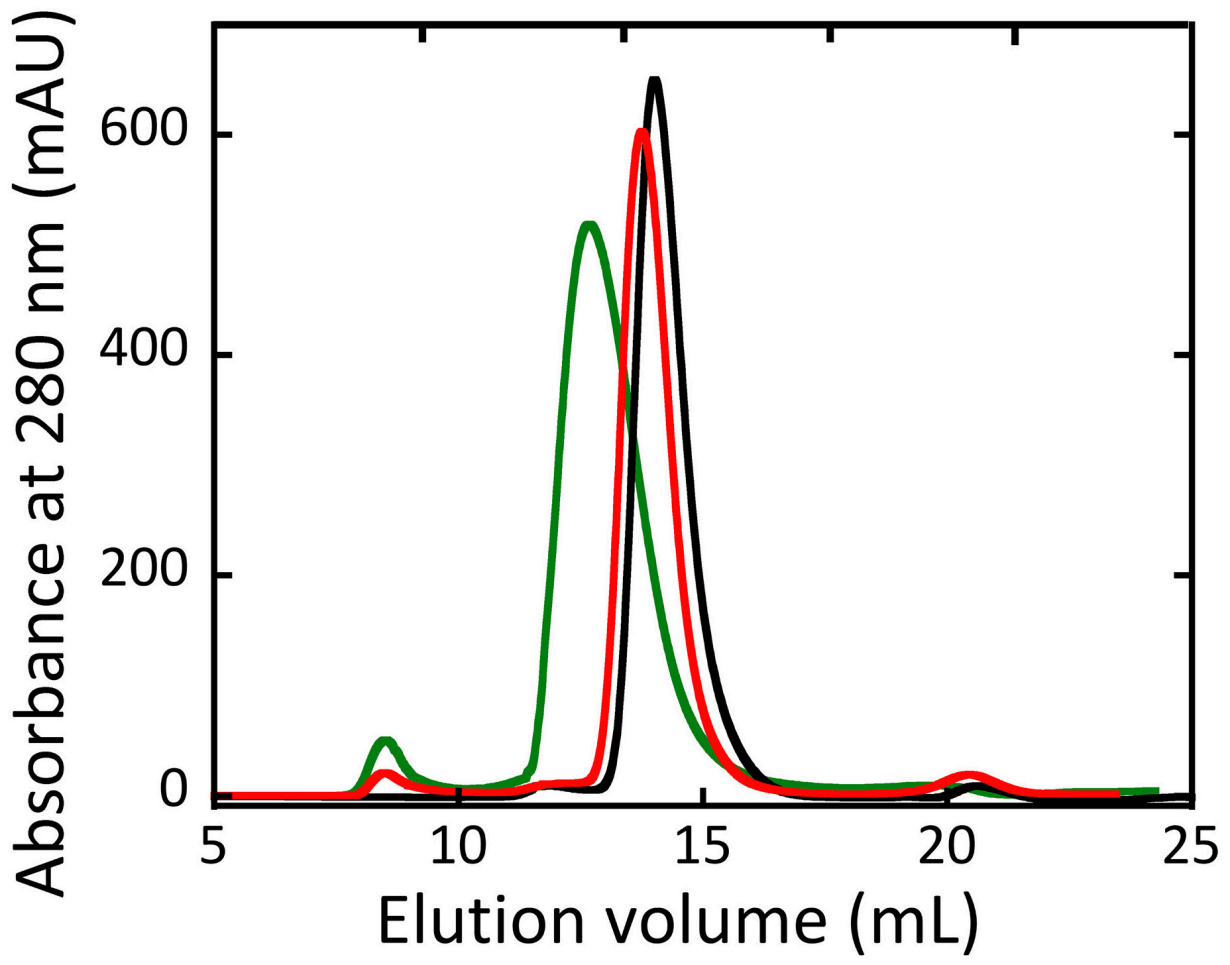
Size-exclusion chromatography elution profiles of wild-type (black), R199Q (red) and R199W (green) hDAAO holoenzymes (5 mg /mL protein concentration). From (Cappelletti et al., 2015).

Size exclusion chromatography studies indicated that hDAAO oligomeric state was unaffected by the G183R substitution (Murtas et al., 2017b). On the other hand, the holoenzyme form of R199W hDAAO was found to be tetrameric, at concentration ≥ 1 mg/mL. Moreover, at lower concentrations, both the holo- and the apoprotein forms of the R199W and R199Q variants showed a chromatographic profile in-between a dimeric and a tetrameric state, suggesting that they assumed a less tightly packed conformation compared to the wild-type hDAAO (Figure 6, Cappelletti et al., 2015).

### Cellular studies

In Cos-7 cells expressing R199W hDAAO (following transient transfection), the inactive variant, similarly to the wild-type enzyme, was shown to be correctly targeted to peroxisomes, where it forms heterodimers with the endogenously expressed wild-type protein. This results in a significant reduction of the enzyme activity, suggesting a potential dominant negative effect of the fALS associated mutation (Mitchell et al., 2010). Accordingly, U87 human glioblastoma cells clones stably expressing G183R, R199W and R199Q hDAAO variants as chimeric protein with the N-terminal region fused with the enhanced yellow fluorescent protein (EYFP), showed significantly increased D-Ser cellular concentrations. In particular, a 7-fold increase in D-Ser levels was observed in cells expressing the G183R variant compared to cells expressing the wild-type protein (Table 5), suggesting that the availability of the neuromodulator at the synapses may be also affected (Cappelletti et al., 2015; Murtas et al., 2017b). Worthy of note, the expression of R199W and R199Q hDAAO (as well as the wild-type enzyme) did not alter U87 cells viability (Cappelletti et al., 2015), as was instead apparent in motor neurons (Mitchell et al., 2010). Similar results were obtained in cell clones expressing the G183R variant (Murtas et al., 2017b). On the other hand, U87 cells transiently expressing G331V hDAAO showed significantly reduced cells viability. Accordingly, isolating stable cell clones overexpressing this hDAAO variant was not feasibile (the transfected cells did not survive to the selection procedure) and cellular studies were performed in transiently transfected cells (Caldinelli et al., 2013). The observed effect was accompained by the formation of consistent amount of the overexpressed protein aggregates and a significant increase in apoptosis, as assessed by caspase activity detection (Caldinelli et al., 2013). Intriguingly, both the G183R and G331V hDAAO variants appeared to be partially mistargeted within the U87 transfected cells: the two variants formed extraperoxisomal protein aggregates, which largely colocalized with ubiquitin (Caldinelli et al., 2013; Murtas et al., 2017b). Both the low solubility and the cytosolic localization of G331V hDAAO, which was supposed to be active (thus to oxidize cytosolic D-Ser producing cytotoxic H_2_O_2_), might explain the observed low cell viability (Caldinelli et al., 2013). Notably, the overexpression of the inactive G183R hDAAO did not enhance apoptosis (Murtas et al., 2017b).

**Table 5:** Effect of the expression of EYFP-hDAAO on the cellular concentrations of serine enantiomers.

**Stably transfected cells**							**Transiently transfected cells**			
							**Time upon transfection**			
							**24 h**		**48 h**	
**hDAAO variants**	**Wild-type**^a^	**G183R**^b^	**R199Q**^a^	**R199W**^a^	**D31H**^c^	**R279A**^c^	**Wild-type**^a^	**W209R**^a^	**Wild-type**^a^	**W209R**^a^
D-Serine (pmol)	1.5 ± 0.3	13.3 ± 1.4	5.9 ± 0.7	2.9 ± 0.9	0.59 ± 0.2	0.38 ± 0.3	12.4 ± 0.6	9.0 ± 1.0	7.1 ± 0.5	5.7 ± 0.01
L-Serine (pmol)	72.8 ± 27.2	45.0 ± 1.6	153 ± 18.4	84.1 ± 18.3	53.7 ± 10.1	28.8 ± 10.8	203 ± 7.0	207 ± 0.8	111 ± 1.7	119 ± 10.3
D-/(D+L)-Serine (%)	2.2 ± 0.5	28.2 ± 8.3	3.7 ± 0.01	3.3 ± 0.3	1.2 ± 0.6	1.2 ± 0.6	5.8 ± 0.4	4.2 ± 0.06	6.0 ± 0.3	4.6 ± 0.04

*HPLC analyses were performed on U87 human glioblastoma cells stably or transiently expressing the different hDAAO variants*.

a *Cappelletti et al., 2015*;

b *Murtas et al., 2017b*;

c *Caldinelli et al., 2013*.

### Hyperactive hDAAO variants potentially involved in psychiatric disorders

Beside the reported inactivating mutations, other amino acidic substitutions were identified in hDAAO. The analysis of the hDAAO protein sequences deposited in the databank revealed a discrepancy at positions 31 and 279, which were indicated as “sequence conflicts.” Namely, Asp31 was substituted with an His residue in a sequence reported in the Uniprot database (http://www.uniprot.org/uniprot/P14920), and Ala279, present in the original sequence of the human *DAO* gene cloned from kidney lysates (Momoi et al., 1988), was substituted by an Arg in the subsequently deposited sequences. To date, it is still unclear whether these differences are due to polymorphisms or sequencing/cloning artifacts. On the other hand, the W209R is a substitution encoded by a confirmed SNP (rs111347906) reported in the database, but currently not associated with a human disease (Cappelletti et al., 2015).

Worthy of note, all the resulting hDAAO substitutions (D31H, R279A, and W209R) were found to exert an opposite effect on the enzyme functionality compared to the ones discussed above: they yielded to hyperactive protein variants (Caldinelli et al., 2013; Cappelletti et al., 2015). The characterization of these protein variants prompted our group to propose their potential involvement in the onset of psychiatric disorders such as schizophrenia: an excessive degradation of D-Ser caused by an abnormal increase of hDAAO enzymatic activity might result into a significant reduced availability of the neuromodulator at the synapses and the consequent hypofunction of NMDAR. This represents a molecular mechanism that could explain many symptomatic features of schizophrenia (Coyle et al., 2003; Coyle, 2006; Stone and Pilowsky, 2007; Pollegioni and Sacchi, 2010).

**Figure 7:**
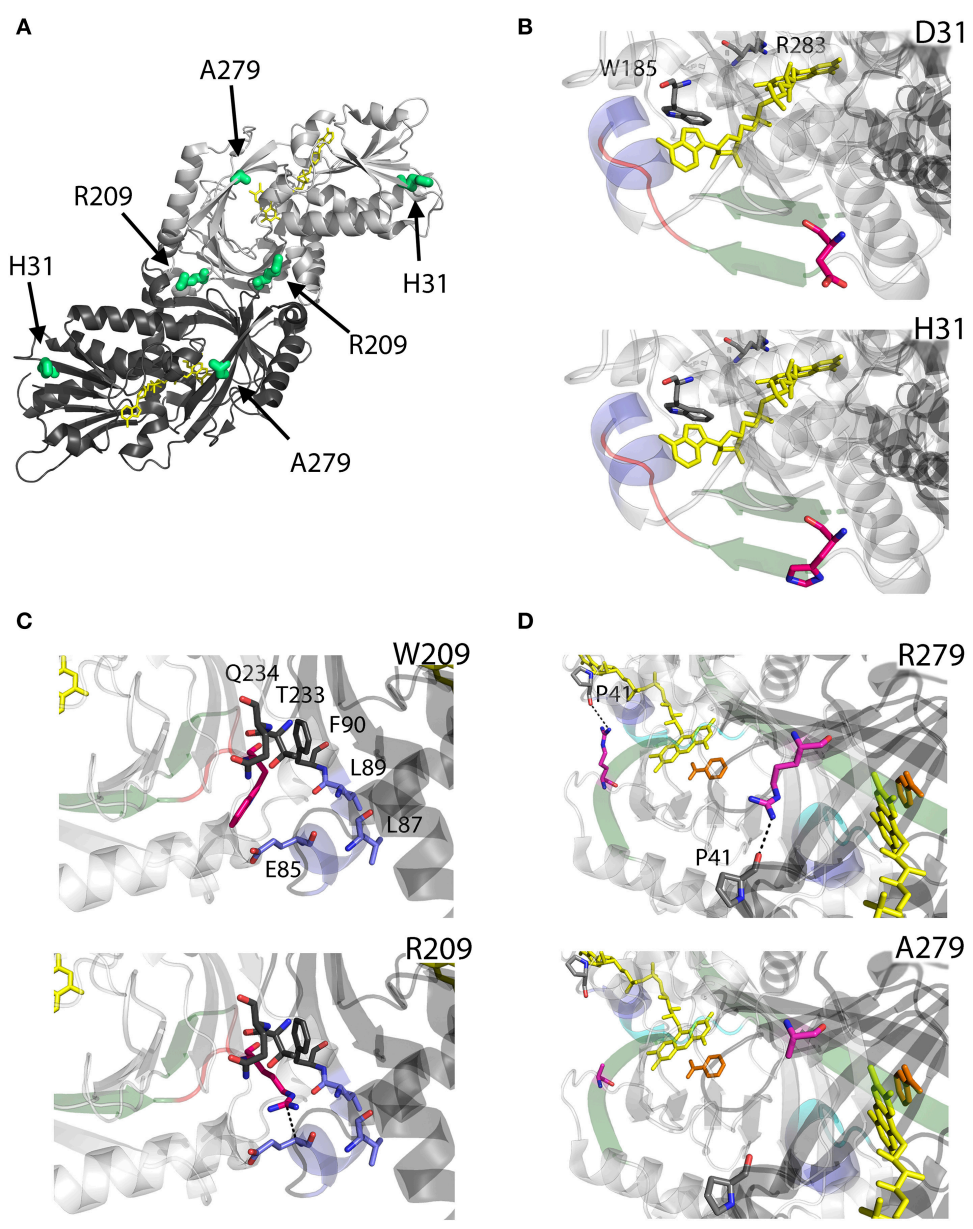
Structural models of hDAAO hyperactive variants. **(A)** Location of the point mutations on the protein dimeric structure (PDB code: 2DU8; Kawazoe et al., 2006). Substituted residues encoded by SNPs are depicted as thick sticks in green, while FAD cofactor is reported in yellow. **(B–D)** Details of the protein microenvironment surrounding at the mutated residues in the different hDAAO variants. The substituted residues are shown as sticks in magenta. The surrounding residues within a distance of 4 Å are shown as sticks in gray. Loops, α-helices, and β-sheets indicated in the text are shown as cartoon in red, blue, and green, respectively. The substrate-competitive inhibitor benzoate and the FAD cofactor are shown as sticks in orange and yellow, respectively. The VAAGL sequence is shown as a cartoon in light blue in **(D)**. H-bond interactions are reported as black dashed line. Figure were prepared with PyMol. Adapted from Caldinelli et al. (2013); Cappelletti et al. (2015).

D31 is a non-conserved residue (Figure 3) located at the β-strand β2 on the protein surface, far from both the substrate and the flavin binding sites, as well as from the dimerization interface (Figure 7A). For this reason, the effect of the D31H substitution was explained in terms of a long-range interaction affecting the overall protein conformation and propagating from the site of the substitutions to other regions in the structure (Caldinelli et al., 2013). Nonetheless, it is possible to speculate that conformational alterations in this position might involve the loop between the β6 and the α8, which central region is close to the nucleotide moiety of the FAD cofactor (Figure 7B).

Conversely, W209 is a well-conserved residue in higher organisms (Figure 3). It is positioned in the loop between β-strands β8-β9 (close to α-helix α4), at the monomers interface (Figure 7A). However, this residue is not involved in relevant hydrophobic interactions with other residues on the surface of the facing subunit: the loss of the indole portion of the tryptophan side chain likely induces minimal modifications of the interaction energy of the hDAAO monomers. Moreover, the positive charge provided by the W209R substitution might result in an additional electrostatic interaction with the negatively charged side chain of E85 of the facing subunit (Figure 7C), de facto stabilizing the dimeric structure (Cappelletti et al., 2015).

R279 is a further non-conserved residue (Figure 3), located on the β-strand β12, which is involved in a double interaction: (i) it contacts the FAD binding site (it is H-bonded with the carbonyl oxygen of P41, close to the α-helix α2 in the region containing the VAAGL motif, which has been proposed to play an important role in determining the affinity of hDAAO for the cofactor; Kawazoe et al., 2006), (ii) contributes to the definition of the interface between the two dimers (Figure 7D). The R279A mutation could affect the flavoenzyme binding affinity for the cofactor by altering the conformation of the VAAGL peptide, directly through the loss of the interaction with P41, or indirectly by a general rearrangement of the β12-α2 interface region (Caldinelli et al., 2013).

### Expression *E. coli* and biochemical properties

Differently to the inactivating hDAAO substitutions, D31H and R279A variants were successfully overexpressed in *E. coli* and purified to homogeneity by using the same conditions set up for the wild-type protein (Table 2; Caldinelli et al., 2013). On the other hand, the fermentation conditions of the W209R variant required further optimization to obtain a final amount of protein similar to the wild-type one (Table 2; Cappelletti et al., 2015).

All these hDAAO variants retained the specific activity of the wild-type flavoenzyme (Table 2; Caldinelli et al., 2013; Cappelletti et al., 2015). They showed a significantly higher affinity for the flavin cofactor (up to 9-fold for R279A hDAAO; see Table 4), thus inducing an increase of the amount of the active holoenzyme species in solution (Caldinelli et al., 2013; Cappelletti et al., 2015). For the D31H and R279A hDAAO variants an improved catalytic efficiency was apparent, due to a decrease in the K_m_ value for D-Ser and a significant increase in the k_cat_ values for D-Ala (Table 3). The W209R hDAAO variant showed an increase in the apparent k_cat_ on both the substrates (Table 3). Notably, this latter variant was more active than the wild-type hDAAO when 0.3 mM D-Ser and 5 μM FAD were present in the reaction mixture, i.e., under conditions reproducing physiological ones (Cappelletti et al., 2015).

For all hyperactive variants, no substantial alteration in inhibitors binding affinity was observed (Table 4; Caldinelli et al., 2013; Cappelletti et al., 2015). Furthermore, all variants retained the ability to interact with the hDAAO binding partner pLG72 (Caldinelli et al., 2013; Cappelletti et al., 2015).

### Alterations in protein conformation

**Figure 8:**
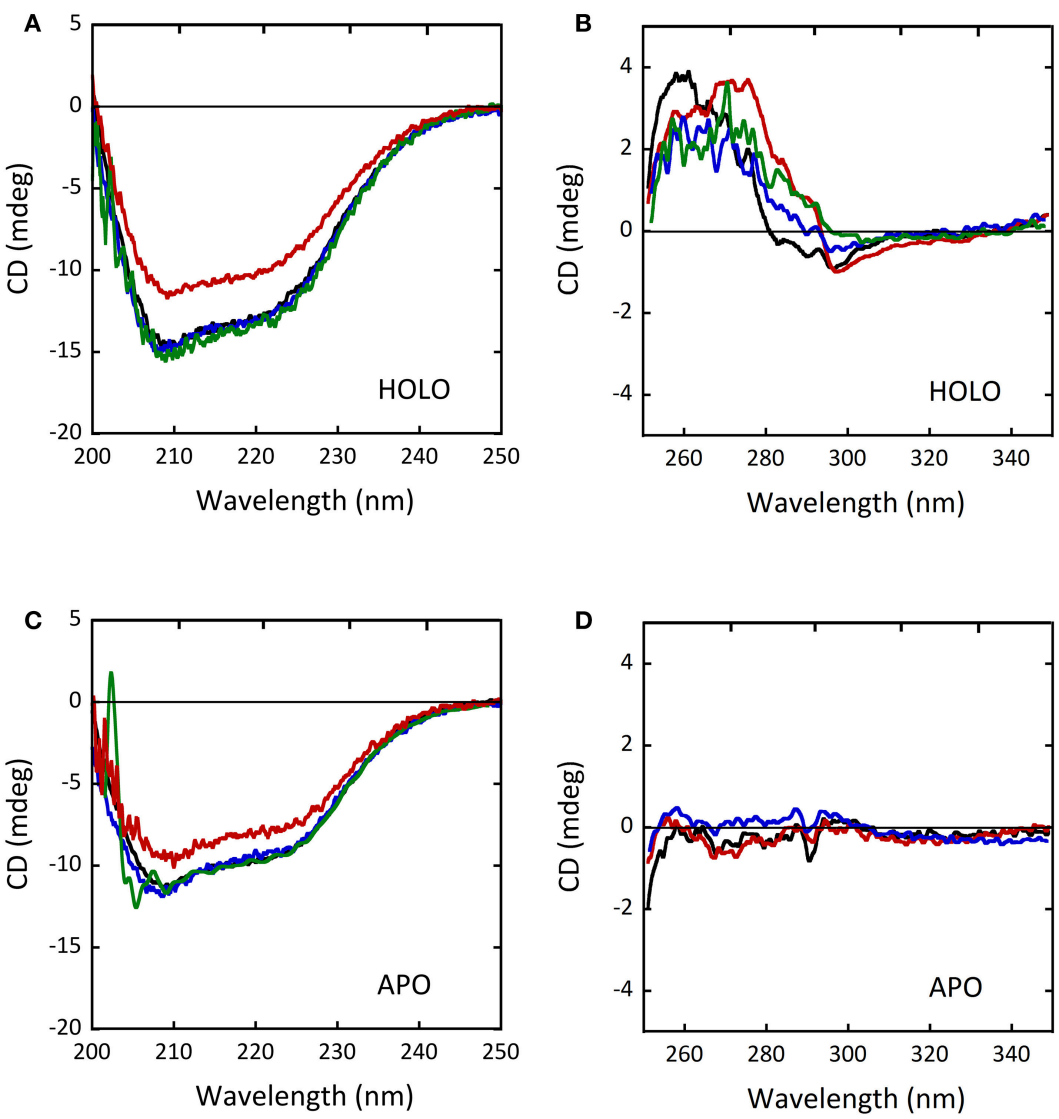
Comparison of CD spectra of wild-type (black), D31H (blue), R279A (green) and W209R (red) hDAAO variants. **(A,C)** Far-UV CD spectra of **(A)** the holoenzyme and **(C)** the apoprotein forms of hDAAO variants (0.1 mg/mL). **(B,D)** Near-UV CD spectra of **(B)** the holoenzyme and **(D)** the apoprotein forms of hDAAO variants (0.4 mg/mL). From (Caldinelli et al., 2013; Cappelletti et al., 2015).

The CD spectra of the holoenzyme and apoprotein forms of the D31H and R279A variants appeared unaltered compared to wild-type-DAAO (Figures 8A–D), indicating that the introduced substitutions did not significantly affect the protein conformation (Caldinelli et al., 2013). On the other hand, differences in far-UV and near-UV CD spectra for the W209R variant were reported (Cappelletti et al., 2015), pointing a decreased α-helix content in the holoenzyme form (~31% of vs. 37% of wild-type protein; Figures 8A,C; Cappelletti et al., 2015). No alterations of the oligomerization state were instead observed (Caldinelli et al., 2013; Cappelletti et al., 2015).

### Cellular studies

In transiently transfected U87 cells the D31H and R279A hDAAO variants fused with EYFP were correctly targeted: the time course of subcellular compartimentalization closely resembled that observed for the wild-type enzyme, and 72 h after transfection they were largely localized to peroxisomes. According to the biochemical characterization, HPLC analysis performed on U87 cell clones stably expressing similar levels of D31H, R279A, or wild-type hDAAO showed that the decrease in D-Ser cellular level was significantly higher in cells expressing the hyperactive variants compared to hDAAO wild-type expressing one (Table 5). This evidence was used to further substantiate the proposed role of hDAAO on D-serine levels related to schizophrenia onset (Caldinelli et al., 2013).

On the other hand, isolating U87 cell clones stably expressing the highly active W209R hDAAO proved to be unfeasible, since its expression was shown to significantly affect cells viability: a massive cell death was reported during the clone selection procedure (Cappelletti et al., 2015). Analyses of D-Ser cellular content were therefore performed on transiently transfected cells. As observed for the other hyperactive variants, W209R hDAAO expression resulted in strongly reduced D-Ser cellular levels compared to control cells expressing the wild-type enzyme (Table 5; Cappelletti et al., 2015). In this case however, an excessive accumulation of H_2_O_2_ produced by the hDAAO catalyzed reaction was suggested to represent the factor affecting cell viability after transfection (see above).

## Conclusions

Structural and functional studies have highlighted the peculiar biochemical properties of hDAAO (Molla et al., 2006; Sacchi et al., 2008, 2012; Caldinelli et al., 2010; Pollegioni and Sacchi, 2010; Cappelletti et al., 2014; Murtas et al., 2017a). It only weakly binds the FAD cofactor, shows a stable homodimeric state, and a relatively low activity on its physiological substrates (D-Ser, D-Ala, D-Cys, and D-DOPA). Moreover, hDAAO activity and half-life appears to be specifically modulated by the interaction with the regulatory protein pLG72 (Sacchi et al., 2008, 2011; Cappelletti et al., 2014; Pollegioni et al., 2018) and bassoon (Popiolek et al., 2011). The flavoenzyme activity must be finely tuned to fulfill what is thought to be its main relevant function: controlling D-Ser concentration in the brain. Altered levels of the neuromodulator may affect NMDAR functions and receptor-related processes involved in cognitive ability such learning and memory (Collingridge et al., 2013; Morris, 2013).

Dysregulation in D-Ser signaling, potentially due to altered DAAO activity, has been implicated in the NMDAR dysfunctions observed in various diseases, such as familial and sporadic ALS (Mitchell et al., 2010; Paul and de Belleroche, 2012), AD (Tannenberg et al., 2004; Billard, 2008; Madeira et al., 2015) schizophrenia (Verrall et al., 2007; Madeira et al., 2008; Habl et al., 2009) and chronic pain (Zhao et al., 2010; Gong et al., 2011). Accordingly, it is of great relevance to biochemically characterize the hDAAO variants reported in protein or SNP databases, especially in the case that the introduced amino acidic substitutions significantly affect the enzyme functional properties. In this regard, hDAAO variants can be basically grouped into two classes: inactive and hyperactive.

G183R, R199W, and R199Q variants are characterized by significantly reduced or fully abolished (in the case of G183R hDAAO) enzymatic activity. They show impaired ligand binding properties due to local or general perturbation in the protein conformation (Cappelletti et al., 2015). In G183R hDAAO variant changes in secondary structure elements likely induce variations in the conformation of the FAD binding domain negatively affecting the correct binding of the cofactor (Murtas et al., 2017b). On the other hand, large modifications affecting the tertiary structure of R199W and R199Q hDAAO variants deeply impact on their catalytic efficiency (Cappelletti et al., 2015). Differently, G331V substitution affects the enzyme activity and stability because the induced changes in the C-terminal α-helix promote protein aggregation, strongly reducing the variant solubility (Caldinelli et al., 2013). On the other hand, in D31H, W209R and R279A hDAAO variants the substitutions (and eventually the induced conformational perturbations) have an opposite effect. These protein variants show slightly to significantly improved ligand binding affinities and catalytic efficiency. In particular, W209R hDAAO shows a 2-fold higher turnover number since the substitution appears to facilitate the product release from the reoxidized enzyme after the catalysis (Cappelletti et al., 2015).

Most importantly, all the reviewed hDAAO variants have been shown to directly and significantly impact on D-Ser cellular levels when they are overexpressed in a U87 cell line (Caldinelli et al., 2013; Cappelletti et al., 2015; Murtas et al., 2017b): this finding confirms their potential significance in pathological conditions where the accumulation or the excessive degradation of their physiological substrates is an important feature. It has been also highlighted that hDAAO variants aggregation and mislocation, and/or the induced overproduction of H_2_O_2_ (in or outside the peroxisomes), might represent important factors significantly affecting cell viability and making the effect of the expression of the different variants even more complex.

Thus, the expression of hDAAO inactive variants in cohorts of individual, as proved for R199W one, might confer susceptibility to neurodegenerative disorders such as ALS and AD, due to an abnormal accumulation of D-Ser which, when associated to elevated glutamate levels, could lead to excitotoxicity related to the hyper-activation of NMDAR. Differently, hyperactive variants might induce a deficit in NMDAR-mediated transmission due to a decreased availability of D-Ser at the synapses and the hypofunction of the receptor, a condition that in turn has been postulated to be involved in schizophrenia (and eventually AD) onset. Their expression would also be significant in individuals affected by, or sensitive to, chronic pain. The overproduction of H_2_O_2_ by hDAAO hyperactive variants (together with the exaggerated depletion of D-Ser) may contribute to the elucidation of the molecular mechanism of the central sensitization typical of this neurological condition.

We are aware that, despite the key role played by hDAAO in some important physiological processes, much remains to be unravel concerning the modulation of its functional properties. The ongoing characterization of variants corresponding to confirmed SNPs in the database will deepen our understanding of the structure/function relationships of this enzyme, and of the effect of their alteration in pathological conditions, providing effective tools to propose and design novel and more effective therapeutic treatments.

Moreover, the potential of the novel genome editing tool based on the clustered regularly interspaced short palindromic repeats (CRISPR)/CRISPR-associated protein-9 nuclease (CRISPR/Cas9) has been recently explored for modeling neurodegenerative diseases which pathomechanisms that have not yet been fully uncovered, including ALS (Kruminis-Kaszkiel et al., 2018). Indeed, both a zebrafish (Armstrong et al., 2016) and a mouse (Liu et al., 2017) ALS models have been developed by using this genome engineering technology to introduce patients-specific mutations in genes associated to the disease. Therefore, the possibility to develop animal models carrying DAAO selected activating or inactivating mutations might represent an invaluable system to investigate further the molecular mechanisms involved in pathological conditions, ranging from neuropsychiatric to neurodegenerative disorders, in which dysregulation of D-Ser metabolism likely play a key role.

## Author contributions

SS designed the review. All authors analyzed the literature and wrote the manuscript.

### Conflict of interest statement

The authors declare that the research was conducted in the absence of any commercial or financial relationships that could be construed as a potential conflict of interest.

## Abbreviations

DAAO: D-amino acid oxidase
hDAAO: human DAAO
D-AAs: D-amino acids
NMDAR: N-methyl-D-aspartate type glutamate receptor
SNP: single nucleotide polymorphism
CBIO: 5-chloro-benzo[d]isoxazol-3-ol
CNS: central nervous system
ROS: reactive oxygen species.
